# Evaluation of the multimodal DELTA therapy for adolescents with substance use disorders: an exploratory pilot trial

**DOI:** 10.3389/fpsyt.2023.1284342

**Published:** 2024-01-03

**Authors:** Lukas A. Basedow, Soeren Kuitunen-Paul, Melina F. Wiedmann, Veit Roessner, Yulia Golub

**Affiliations:** ^1^Division of Clinical Psychology and Psychotherapy, Philipps-University Marburg, Marburg, Germany; ^2^Chair of Clinical Psychology and Psychotherapy, TU Chemnitz, Chemnitz, Germany; ^3^Department of Child and Adolescent Psychiatry, Technische Universität Dresden, Dresden, Germany; ^4^Department of Child and Adolescent Psychiatry, TU Dresden, Dresden, Germany; ^5^Department of Child and Adolescent Psychiatry, University of Oldenburg, Oldenburg, Germany

**Keywords:** substance use, addiction, drug abuse, therapy, adolescence

## Abstract

**Background:**

In order to address the lack of manualized treatment programs for adolescents with substance use disorders (SUDs), we developed a manualized group intervention (DELTA). DELTA focusses on substance use reduction and abstinence as well as alleviation of SUD symptoms via additional modules for co-occurring disorders. The goal of this exploratory trial was to assess if DELTA can be conducted in adolescent SUD patients and if participation is related to reductions in substance use, SUD-related problems, and further psychopathologies.

**Method:**

We recruited adolescents at a psychiatric outpatient unit, which were then allocated to either DELTA intervention group (*N* = 85) or to a waiting-list control group (WL, *N* = 61) based on parental decision to start a therapy or not. Self-report measures were used as primary outcomes (substance use via interview, use-related problems via DUDIT—Drug Use Disorder Identification Test) and secondary outcomes (psychopathologies via YSR—Youth Self Report). *T*-tests and Pearson correlations were used to analyze between-group differences across time.

**Results:**

On average, participants attended *M* = 7.7 (SD = 5.1) of the 16 sessions. Substance use and use-related problems regarding all substances but nicotine was decreased after the intervention, with small to medium not significant effects in favor of DELTA. Self-reported psychopathologies were also reduced at follow-up, with non-significant advantages for DELTA.

**Conclusion:**

DELTA showed small effects on SUD-related and depression-related variables. However, the interpretation is limited by the small sample size. Nonetheless, the DELTA intervention is viable in SUD outpatient treatment and will be further evaluated.

**Clinical trial registration**: The study was registered at clinicaltrials.gov under NCT03444974. Registered February, 26th 2018 (https://clinicaltrials.gov/ct2/show/NCT03444974).

## 1 Introduction

About 2–10% of adolescent substance users develop a substance use disorder (SUD) (1). Previous research from our group showed that cannabis use disorder (CUD) is the most frequent SUD in adolescent outpatient treatment settings (2). Most adolescent SUD patients also present with multiple SUDs and have at least one co-occurring disorder (2–5). Treatment options for adolescent SUDs in Germany are mostly limited to inpatient detoxification and outpatient drug counseling (6, 7). Those few programs specifically designed for adolescents provide guidance only for the treatment of specific SUDs (8), require a setting that involves stays at the clinic for up to 8 h per day (9) or are not available in German (9, 10). Available treatment guidelines for SUDs recommend that interventions should include the treatment of co-occurring mental disorders, given that those are widespread in adolescent SUD (6, 11–13) and have been repeatedly shown to influence the therapy outcomes (14, 15). Given this lack of adolescent-specific, integrative programs, we have developed the “Dresden multimodal therapy for adolescents with chronic substance use” (German abbreviation: DELTA) (16), a 16-week group and individual therapy for SUD including modules on psychopathologies.

### 1.1 Hypotheses

Our first hypothesis was that DELTA is associated with a stronger reduction in SUD severity and substance use as compared to a waiting-list control group (WL). A second hypothesis was that DELTA is associated with a stronger decrease in symptoms of co-occurring psychopathologies. Reductions in nicotine use, psychotic prodromal symptoms and attention problems are additionally explored, although DELTA does not focus on these problems.

## 2 Methods

### 2.1 Procedures and design

**Figure 1:**
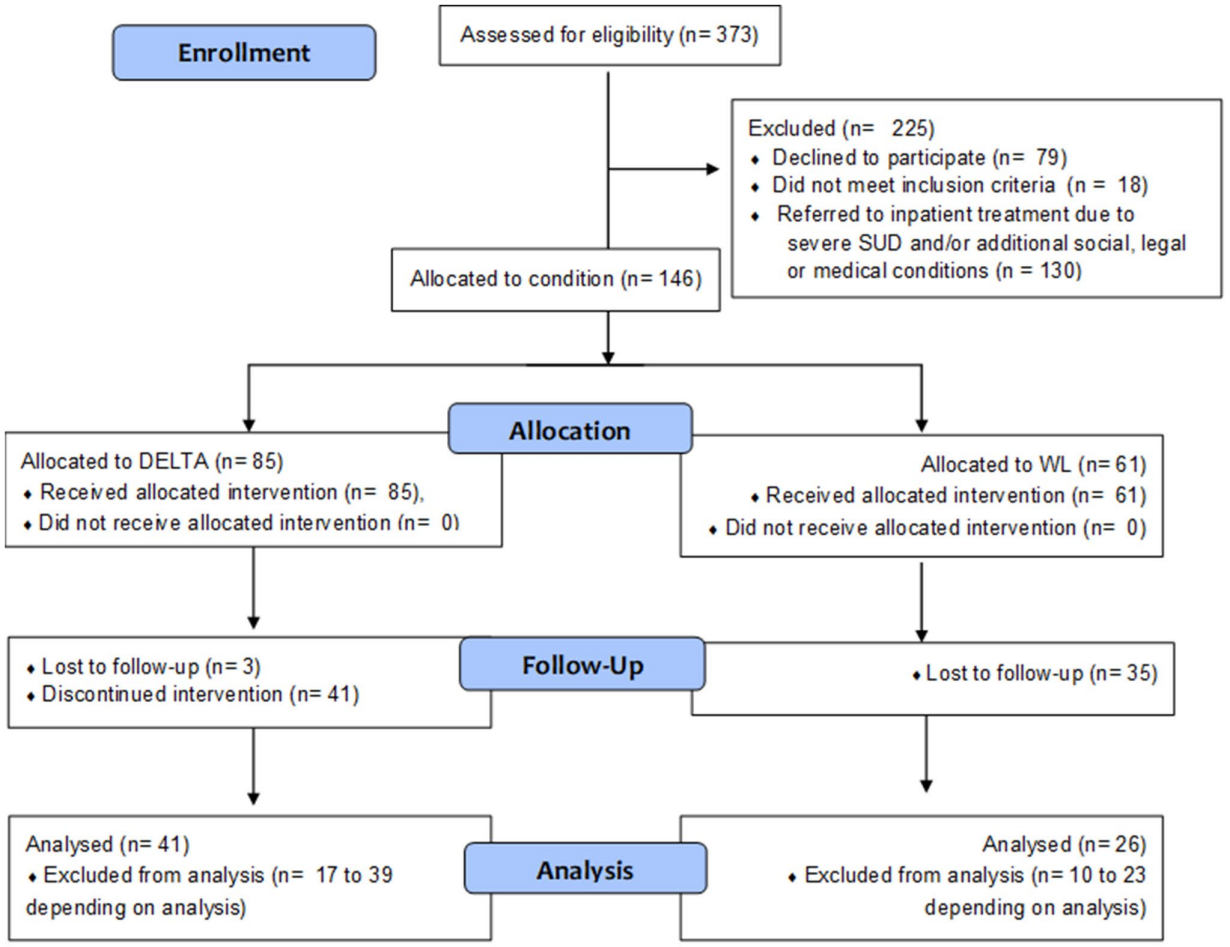
Study flow diagram.

Participants were recruited between November 2017 and September 2021 from our outpatient department for adolescent substance abuse, and three cooperating youth welfare institutions. For an overview of study flow, see Figure 1. We included adolescents qualifying for any SUD according to ICD-10 criteria, aged between 12.00 and 18.99 years, applying the following exclusion criteria: Pre-existing neurological diseases; diseases of the central nervous system, the adrenal, pituitary gland or hypothalamus; intelligence quotient <70; acute viral diseases; therapeutic decision that inpatient detoxification is warranted.

Participants from the outpatient department received a treatment recommendation after a multidisciplinary team of clinical expert reviewed their case. Those who agreed to participate but did not want to join the DELTA intervention were assigned to the WL condition (see Section 2.4). Thus, based on the pragmatic concerns of clinical care, true randomization was not possible. Participants living in youth welfare institutions were cluster-randomized to either WL or DELTA. In accordance with recommendations for clinical trials in small populations (1), WL participants in youth welfare institutions received DELTA after a 16-week waiting period. Therefore, this evaluation study enabled WL controls with a delayed intervention.

First, baseline assessments (BL) were conducted including questionnaires, structured interviews, as well as cognitive tests and a physical examination. After completion of the DELTA group sessions or passing at least 16 weeks to fulfill the WL condition, a similar follow-up assessment (FU) was conducted. Depending on the availability of participants, the FU measures were taken on average at 26.3 weeks (SD = 6.7 weeks, range = 15.0–53.1 weeks) after BL, i.e., approx. Eight weeks after the last DELTA session.

Both patients and legal guardians agreed to study participation by written consent after a comprehensive verbal and written information. Patients did not receive reimbursements for participation in the group sessions, which were carried out in the outpatient setting. Blinding was not possible for participants or personnel administering the intervention. All procedures were conducted in accordance with the Declaration of Helsinki and were approved by the local Institutional Review Board (EK 66022018), see also clinicaltrials.gov registry entry NCT03444974 and Supplementary Table S1 [Extended CONSORT checklist (2)].

### 2.2 Participants

*N* = 373 adolescents were recruited from one of the outpatient settings. *N* = 294 participants and their legal guardians agreed to participate. From these, *n* = 18 (6.1%) did not meet inclusion criteria and *n* = 130 (44.2%) were referred to inpatient treatment after screening. *N* = 9 WL participants in youth welfare institutions received DELTA following a waiting period, which resulted in a sample comprised of *n* = 146 adolescent SUD patients for analysis. All participants who participated at least in one therapy session were included in the analysis. Participants who discontinued the intervention subsequently did not provide FU data.

### 2.3 Intervention

DELTA (3) involved 16 weekly sessions with 1–2 trained and experienced psychologists leading a group of 3–8 adolescents, lasting 60 min. Additionally, each patient receives up to 8 individual session every 2 weeks (max. 60 min) that may be used to address individual difficulties regarding psychosocial functioning, legal issues, educational career planning, etc. Before the first group session, all participants work out a therapy agreement with the therapist, detailing the rules of participation and the planned date of abstinence. Cognitive-behavioral methods are included to analyze use-related cognitions and behaviors in terms of their preceding stimuli. Another behavioral method consists of applying regular positive reinforcement for active participation in the group. DELTA integrates principles of Motivational Interviewing (4), in a manner that accepts the continuous ambivalence of SUD patients toward abstinence. Additionally, contingency management is used to reward completed homework. While creating DELTA we aimed to integrate the central mechanisms of psychological SUD treatment (5): therapeutic relationship, analysis of reasons for substance use, cooperative goal setting, and skills for dealing with emotional crises.

Participants were formally required to attend all sessions if possible and had to provide a valid explanation for missed meetings. In case two or more meetings were missed without valid explanation, participants were temporally excluded from participating in the sessions. Re-entry into the group sessions was possible after an eight-week break or a consultation with the attending therapist.

### 2.4 Waiting-list condition

Participants in the WL condition in both settings (outpatient and welfare institutions) received treatment as usual, which could include measures such as individual therapy, psychiatric care or referral to inpatient treatment in cases of severe SUD after unsuccessful outpatient intervention. After 16–20 weeks, WL participants were included in the DELTA intervention.

### 2.5 Outcome measures

#### 2.5.1 Substance use problems

The Drug Use Disorders Identification Test (DUDIT) (6) is a self-report instrument that assesses problems related to the use of illicit psychoactive substances. The DUDIT total is calculated by summing the scores on all items, with a maximum score of 44. The DUDIT-C subscale can be calculated from the first four items (7), with a maximum score of 16. The outcome variables from this questionnaire were the change between baseline and follow-up (FU) in DUDIT total and DUDIT-C score.

#### 2.5.2 Substance use

Substance use was assessed via interview by a clinical psychologist. The interview recoded the number of days each substance (nicotine, alcohol, cannabis, methylenedioxymethamphetamine (MDMA), amphetamine, methamphetamine, hallucinogens, opiates, inhalants, or other) was consumed in the past month as well as the average quantity of the substance per use. A general substance use index “QF” for the past month was calculated by multiplying frequency and quantity. Additionally at baseline, the interview included questions about frequency and quantity of substance use averaged across the past year. The main outcome variables of the instrument were the differences between average monthly QF at baseline (referring to past-year use) and at FU (referring to past-month use).

#### 2.5.3 Psychopathologies

Depressiveness was covered using the Beck Depression Inventory II (BDI-II) (8), a self-report questionnaire with 21 items (Likert scale ranging from 0 to 3) resulting in a sum score where larger values equal stronger depressiveness. The Youth Self Report form (YSR), a multidimensional questionnaire with 118 items (Likert scale ranging from 0 to 2), measures a range of different psychopathologies across eight subscales, including those related to depression-related affective symptoms (“YSR anxious/depressive”), depression-related social impairments (“YSR social withdrawal”), attention-deficit disorder-related problems (“YSR attention”), and conduct disorder-related problems (“YSR aggressive” as well as “YSR dissocial”) (9). Psychopathologies related to PTSD are assessed via the UCLA PTSD scale (10), a questionnaire assessing PTSD symptoms in all three symptom clusters. Psychopathologies related to prodromal symptoms of psychoticism are assessed via the Prodromal Questionnaire (PQ16) (14) with its 16 items (true vs. false) summed up to a score, with larger values indicating more symptoms of psychotic prodromal phases.

#### 2.5.4 Life satisfaction

Adolescents rated their global life satisfaction on the Satisfaction with Life Scale, German version (SWLS) (15), a 5-item questionnaire (Likert scale ranging from 1 to 7), with higher scores indicating higher life satisfaction.

All self-report measures were presented in their respective German version.

### 2.6 Descriptive measures

#### 2.6.1 Adherence to therapy

The number of group sessions attended by each patient was used as an indicator for adherence to the therapy, ranging from 1 to 16 sessions.

#### 2.6.2 Therapy content evaluation

A self-designed questionnaire was applied to assess how helpful the participants perceive the group sessions to be. The questionnaire contains 20 items that are related to the contents of the different group sessions and refer to their usefulness in daily life. Each item is rated on a five-point scale (0 Never, 1 Rarely, 2 Sometimes, 3 Often, 4 Always) to indicate how helpful the specific content was for the participant’s daily life.

#### 2.6.3 DSM-5 diagnoses

SUDs as well as co-occurring mental disorders were assessed with the Mini-International Neuropsychiatric Interview for Children and Adolescents (MINI-KID) (16).

#### 2.6.4 Sociodemographic characteristics

Information on patient age and gender were assessed by therapists during the intake meeting in our clinic. Recorded variables were age in years and gender (male/female).

### 2.7 Statistical analysis

Analyses were conducted with IBM SPSS Statistics 27.0. In most questionnaires (DUDIT, BDI-II, PQ16, SWLS), missing values were imputed if 80% or more of the items were answered. For YSR and UCLA PTSD scales, sum scores were used that ignored single item missing values. To investigate the relationship between SUD severity and substance use, group differences in DUDIT, DUDIT-C and QF change were calculated via multiple *t*-tests. To test the second study aim (reducing symptoms of co-occurring disorders) multiple *t*-tests were calculated. A multivariate analysis would have severely limited the number of analyzed cases given that different cases had different missing patterns. In accordance with recommendations for clinical trials in small populations (1), we moved to one-sided significance testing against a predefined α = 0.05. In case of statistical significance, we corrected for the increased chance of false positives due to multiple testing by the Bonferroni correction. Effect sizes were classified according to Cohen (17) into small effects (|*d*| ≥ 0.20), medium effects (|*d*| ≥ 0.50), and large effects (|*d*| ≥ 0.80).

## 3 Results

### 3.1 Baseline group composition

Analyzed participants aged 12.7–18.7 years (*M* = 16.1, SD = 1.2) included 38.4% females, see Table 1. The majority qualified for more than one SUD (57.0%) excluding nicotine use disorder. Both the DELTA intervention group and the WL group were comparable at baseline in terms of demographic and substance use characteristics, except for the proportion of participants recruited from youth welfare institutions being higher in the DELTA group (41% vs. 19%, *p* = 0.006). From *n* = 146 baseline participants, *n* = 67 (45%) were reached for FU, of which *n* = 41 were part of the DELTA intervention group.

**Table 1:** Demographic and substance use characteristics of both groups at baseline.

	Total sample	DELTA group	WL group	Group differences		
	*n* = 146	*n* = 85	*n* = 61	Test statistic (df)	*p*	Effect size (d)
Females, *n* (%)	56 (38%)	35 (41.2%)	21 (34.4%)	*X*^2^ (1) = 0.68	0.408	0.13
Age in years, *M* (SD)	16.1 (1.2)	16.1 (1.2)	16.2 (1.1)	*t* (144) = 0.08	0.465	0.01
Living in youth welfare institutions, *n* (%)	47 (32%)	35 (41%)	12 (19%)	*X*^2^ (1) = 7.52	0.006*	0.46
**Substance use disorders, *n* (%) (*n* = 39 missings)**						
Alcohol	53 (49%)	30 (42%)	23 (62%)	*X*^2^ (1) = 3.60	0.057	0.37
Cannabis	76 (71%)	49 (70%)	27 (72%)	*X*^2^ (1) = 0.10	0.747	0.06
Stimulants	49 (45%)	35 (50%)	14 (37%)	*X*^2^ (1) = 1.44	0.230	0.23
Benzodiazepines	2 (1%)	2 (2%)	0	–	–	–
Opiates	3 (2%)	2 (2%)	1 (2%)	–	–	–
Hallucinogens	1 (<1%)	1 (1%)	0	–	–	–
Inhalants	1 (<1%)	1 (1%)	0	–	–	–
DUDIT score, *M* (SD)	17.8 (10.5)	18.2 (10.5)	17.2 (10.6)	*t* (109) = −0.48	0.314	0.01

*Significant at the 0.05 level.DUDIT, Drug Use Disorders Identification Test; WL, waiting-list condition.

#### 3.1.1 Non-responder analysis

Neither in DELTA nor WL, adolescents reached for FU differed from those lost to FU in terms of gender distribution, age, or DUDIT score (see Supplementary Table S2). However, specifically in the DELTA group, those who were reached for FU had a higher baseline DUDIT score compared to those lost to FU.

### 3.2 Therapy adherence and content evaluation

In the DELTA group, adolescents participated in an average of 7.7 group sessions (SD = 5.1), with 37% (*n* = 32) of them continuing up to the 10th group session. Subjective ratings of how useful the therapy was perceived showed an average rating of *M* = 2.3 (SD = 0.38). That is, the therapy was typically rated as helping “sometimes” in all relevant aspects except for enhancing self-confidence, reducing fear, reducing feelings of helplessness, and increasing knowledge about substance use (see also Supplementary Figure S1). Highest ratings were shown for “reducing conflicts with important others,” for “improving relationships with important others,” and for “increasing control over one’s own substance use behavior.”

### 3.3 Substance use outcomes

**Figure 2:**
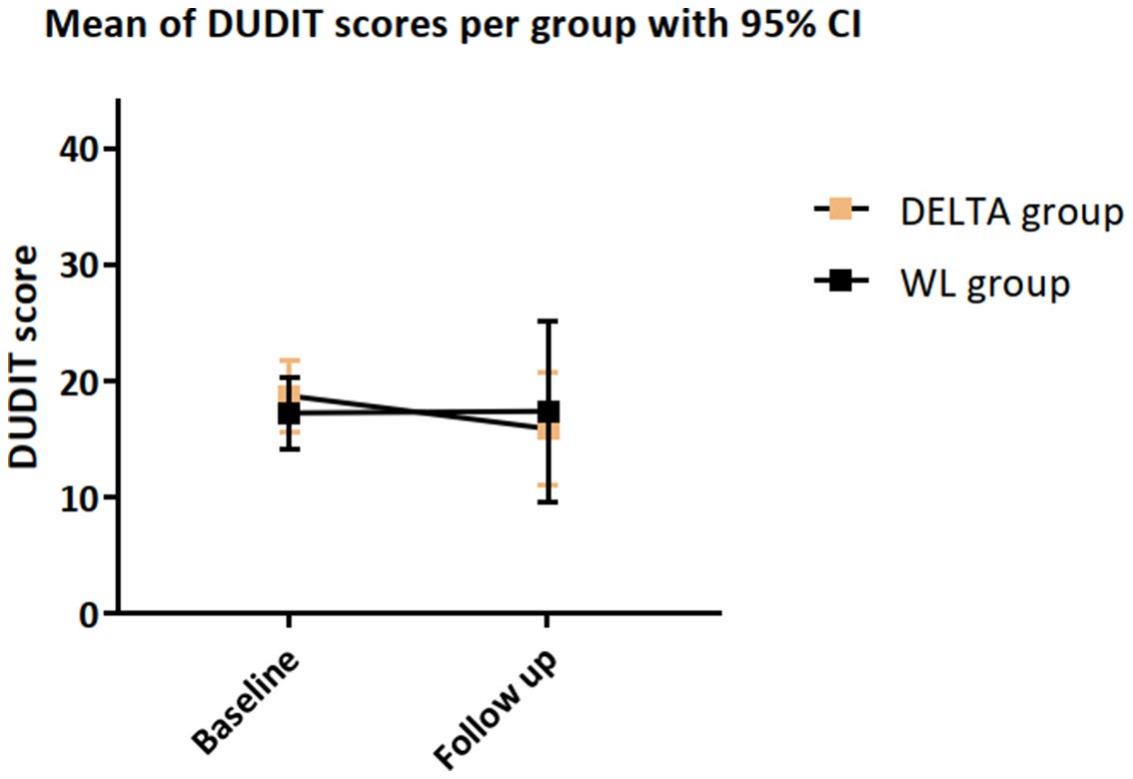
Changes in DUDIT score from baseline to FU, with statistics from *t*-test comparing the change score between the DELTA and WL condition.

We observed a large yet nonsignificant reduction in DUDIT score in the DELTA group in comparison with the WL group with a small effect size, *d* = 0.23 (*p* = 0.295), see Table 2 and Figure 2. In the DELTA group, all QF values decreased between baseline and FU. In the WL group, all QF values except methamphetamine and alcohol reduced as well. The difference between the groups in methamphetamine change was equivalent to a large sized effect (*d* = 1.54, *p* = 0.022), the difference in nicotine, cannabis, and MDMA use change was equivalent to a small effect (all *d* > 0.20, all *p* > 0.17), and the difference in alcohol use change was of a negligible size (*d* = 0.12, *p* = 0.38). The number of attended sessions was strongly associated with amphetamine (*r* = −0.69, *p* = 0.198) and methamphetamine use (*r* = −0.67, *p* = 0.327), moderately associated with nicotine use (*r* = −0.31, *p* = 0.128), alcohol use (*r* = −0.31, *p* = 0.258), social withdrawal (*r* = −0.33, *p* = 0.208), dissocial symptoms (*r* = −0.32, *p* = 0.223) and attentional problems (*r* = −0.36, *p* = 0.163), see Supplementary Table S3.

**Table 2:** Group differences in primary outcomes, with positive effect sizes indicating a bigger reduction in the DELTA group.

	Total sample	DELTA group	WL group	Group differences		
Mean change (from baseline to FU) regarding	*N* total (DELTA:WL)	Δ*M* (SD)	Δ*M* (SD)	Test statistic (df)	*p*_one-sided_ (corrected)	Effect size (*d*)
**Substance use**						
Cigarettes per month	42 (26:16)	−224.5 (356.4)	−123.8 (286.4)	*t* (40) = 0.95	0.173 (0.865)	0.30
Standard drinks alcohol per month	26 (15:11)	−56.9 (223.3)	−80.3 (133.7)	*t* (24) = −0.30	0.380 (0.999)	−0.12
Grams of cannabis per month	28 (18:10)	−60.4 (98.2)	−34.0 (50.6)	*t* (26) = 0.78	0.219 (0.999)	0.31
Number of ecstasy pills per month	13 (8:5)	−6.4 (15.7)	−3.5 (6.5)	*t* (11) = 0.39	0.351 (0.999)	0.22
Grams of methamphetamine per month	11 (4:6)	−61.0 (68.1)	5.0 (12.2)	*t* (8) = 2.38	0.022 (0.110)	1.54
DUDIT-C score	21 (14:7)	−3.4 (6.3)	−3.1 (5.5)	*t* (19) = 0.10	0.460	0.04
**Substance use problems**						
DUDIT score	24 (16:8)	−5.2 (13.4)	−2.4 (8.2)	*t* (22) = 0.54	0.295	0.23

Amphetamine not included because only *n* = 1 in waiting list. *p*-values were not corrected for multiple testing when differences were not statistically significant in the first place. Bonferroni correction was applied for substance use variables (5 comparisons).FU, follow-up; DUDIT, Drug Use Disorders Identification Test. MDMA, 3,4-Methylenedioxy methamphetamine. WL, waiting-list condition.

### 3.4 Psychopathological outcomes

As shown in Table 3, depressiveness decreased over time in both groups, with no differences between groups (*d* = −0.06, *p* = 0.445). Psychopathologies broadly related to depression decreased in the DELTA group while these same psychopathologies increased in the WL group, representing a medium-sized group difference (*d* = 0.54, *p* = 0.180 and *d* = 0.78, *p* = 0.100). Psychopathologies related to conduct disorder decreased in both groups with a small to medium advantage for DELTA (*d* = 0.74, *p* = 0.125, and *d* = 0.22, *p* = 0.348). Psychopathologies related to PTSD did not change meaningfully over time nor did groups differ from each other as seen irrelevant or small effect sizes (*d* = 0.00 to −0.41, *p* = 0.198 to 428). The same pattern was shown for psychopathologies related to psychoticism and schizophrenia (*d* = 0.12, *p* = 0.399). Attention problems decreased in both groups with a small insignificant advantage for WL (*d* = −0.25, *p* = 0.329). Satisfaction with life in general increased in both groups, without differences between DELTA and WL (*d* = −0.15, *p* = 0.364). The number of attended sessions was moderately associated with decreased social withdrawal, decreased attention problems, and decreased dissocial behavior related to conduct disorder (all *r* = −0.32 to −0.36, all *p* = 0.163 to 0.223, see Supplementary Table S3).

**Table 3:** Group differences in secondary outcomes.

Secondary outcome	Related co-occurring disorder/condition	*N*	DELTA group	WL group	Group differences		
	(Prevalence at baseline in the total sample^a^)	(DELTA:WL)	Δ*M* (SD)	Δ*M* (SD)	Test statistic (df)	*p*_one-sided_	Effect size (*d*)
BDI-II sum	Depression (12%, *n* = 6/49)	25 (17:8)	−2.4 (13.3)	−3.2 (14.4)	*t* (23) = −0.14	0.445	−0.06
YSR anxious/depressive	Depression (12%, *n* = 6/49)	20 (16:4)	−3.3 (8.2)	+1.5 (13.1)	*t* (18) = 0.94	0.180	0.54
YSR social withdrawal	Depression (12%, *n* = 6/49)	20 (16:4)	−4.1 (9.4)	+2.5 (5.1)	*t* (18) = 1.39	0.100	0.78
YSR aggressive	Conduct disorder (57%, *n* = 28/49)	19 (16:3)	−4.0 (5.2)	−0.3 (0.5)	*t* (17) = 1.18	0.125	0.74
YSR dissocial	Conduct disorder (57%, *n* = 28/49)	20 (16:4)	−6.3 (7.5)	−4.7 (3.2)	*t* (18) = 0.39	0.348	0.22
UCLA symptoms intrusion	PTSD (22%, *n* = 11/49)	19 (12:7)	0.0 (1.1)	0.0 (0.5)	*t* (17) = –	–	–
UCLA symptoms avoidance	PTSD (22%, *n* = 11/49)	19 (12:7)	+0.3 (1.6)	−0.2 (1.2)	*t* (17) = −0.87	0.198	−0.41
UCLA symptoms hyperarousal	PTSD (22%, *n* = 11/49)	19 (12:7)	+0.2 (1.4)	+0.1 (0.3)	*t* (17) = −0.18	0.428	−0.08
**Exploratory**							
YSR attention	ADD/ADHD (24%, *n* = 12/49)	20 (16:4)	−0.8 (10.4)	−3.5 (8.5)	*t* (18) = −0.45	0.329	−0.25
PQ16 sum	Psychosis (2%, *n* = 1/49)	19 (12:7)	−0.5 (1.3)	−0.4 (1.1)	*t* (17) = 0.26	0.399	0.12
SWLS sum	Life satisfaction	23 (14:9)	+1.5 (7.3)	+0.6 (2.5)	*t* (21) = −0.35	0.364	−0.15

a Disorder diagnoses according to DSM-5 as assessed with the MINI-Kid interview for current disorders. *p*-values were not corrected for multiple testing given since differences were not statistically significant in the first place.

ADD/ADHD, attention-deficit disorder with/without hyperactivity. BDI-II, Beck Depression Inventory II. PQ16, Prodromal Questionnaire. PTSD, post-traumatic stress disorder. SWLS, Satisfaction With Life Scale. UCLA, UCLA PSTD questionnaire. WL, waiting-list condition. YSR, Youth Self Report questionnaire.

## 4 Discussion

We developed the DELTA intervention for adolescents with SUD. This evaluation of the DELTA intervention suggested a favorable effect of DELTA as compared to the WL controls in line with our first hypothesis. Due to a small sample size, statistical significance was lacking in most outcomes despite considerable effect sizes for changes in substance use, less SUD-related problems, and decreased psychopathologies.

In terms of our primary goal, we found a small effect in the reduction of SUD severity in comparison to the WL group. Similar findings of small or inconsistent improvements were reported in studies for SUD in adults (11). Furthermore, we observed a medium sized intervention effect in terms of reduced use of nicotine, cannabis and MDMA at FU, as well as a large effect for reduction of methamphetamine use. Given the exceptional size of the methamphetamine-related effect, a direct replication of these effects seems unlikely. However, a similar study to ours in adolescents with CUD showed a 7-point decrease of CUD severity compared to our finding of a 5-point decrease across substances (12). It should be noted that these differences might underestimate the efficacy of DELTA given that participants attended only half of the group sessions on average. This low attendance rate might be driven by the inclusion of patients who used amphetamine or methamphetamine, both of which were negatively associated with attendance. While this suboptimal adherence rate is similar to rates observed in other group therapies for SUD (13, 18), an increased adherence might lead to even stronger intervention effects.

Additionally, we observed a small effect on the reduction of the depressive symptoms, understanding and influencing aversive emotions, and promoting prosocial behavior. Another study on manualized treatment of CUD showed an enduring relationship between decreasing cannabis use and decreasing depression among adolescents lasting for 9-months after receiving psychosocial interventions for CUD (19). These findings indicate that a treatment involving a reduction of substance use and SUD symptoms, such as DELTA, might also help to reduce secondary psychopathologies such as depressive symptoms (20, 21). On the other hand, these results might indicate that participants with secondary psychopathologies might find it more difficult to stay in these kind of treatment programs.

Finally, results showed that antisocial behavior might be decreased by DELTA. Conduct disorder is prevalent in adolescents with SUD in general and specifically in methamphetamine users (22), and was present in more than half of our participants Reducing the underlying condition is a beneficial side effect, while it remains unclear if DELTA is the specific reason for this reduction, or if those whose conduct disorder problems already are in decline are more likely to engage in outpatient therapies such as DELTA.

In addition to these suggestions of specific effects of the DELTA intervention, we found that some domains of comorbid psychopathology were not affected. Specifically, PTSD symptoms, prodromal psychotic symptoms and symptoms of attention deficits showed only small to negligible changes in both groups. These results imply that SUD-focused treatments like DELTA or treatments as usual are not sufficient in the treatment of these comorbid conditions. This is in line with current treatment recommendations that suggest that all present conditions should receive specific attention in therapeutic settings (23). Additionally, this result might support the notion that some comorbid disorders (e.g., PTSD) are causally involved in the development of a SUD, specifically in the form of an underlying disorder that is self-medicated with substance use (24, 25).

Finally, an implementation of DELTA into an adolescent health care system would be a resource-intensive undertaking. Regular group sessions as well as individual therapy and group sessions for parents require a large amount of organizational and staff resources, if implemented anew. However, most clinical settings for adolescent SUDs already offer a similar structure of combined group and individual therapeutic sessions (26, 27). Additionally, the literature already shows that inclusion of family members might improve SUD outcomes (27, 28). Therefore, while implementing the DETLA program without existing structures might be only possible in limited cases, adapting existing settings to implement DELTA might be a more efficient approach. Importantly, the DELTA intervention, if reproduced successfully, might provide an important guideline for designing treatment settings that include patients with heterogeneous substance use patterns, heterogeneous additional psychopathologies and allow for outpatient treatment instead of highly restrictive and structured inpatient settings.

### 4.1 Limitations

The small number of patients required strategies for analyzing small samples in clinical trials (1). However, strategies such as one-sided testing, combination of FU assessments across a larger period of time, and re-allocating participants may reduce the internal validity of the study. The small sample may also limit the heterogeneity of the sample as well as decrease the possibility to find significant effects of additional factors. Further, we did not systematically collect data regarding the reason for missing outcome data, which does not allow us to make inferences regarding the reason for drop-out and thus we may miss important sources of biases in our analyses. Additionally, the small sample size prohibited us from performing subgroup analysis, e.g., regarding outpatient settings or type of substance. This would have been an especially important analysis since the DELTA group showed a higher group of participants living in welfare institutions and thus participants might have had less access to substances, artificially increasing the psychological effect size of the intervention. Nevertheless, our study is one of the first ones to investigate a group-based manualized treatment for adolescents with heterogeneous SUDs. As in other multimodal interventions, it is difficult to distinguish which modules contribute to treatment effects. Furthermore, adolescent patients with SUDs often participate in treatment only reluctantly, as reflected in low session attendance, which decreases the possibility of detecting meaningful effects. Additionally, participants who declined participation in the DELTA group might be less motivated to receive and participate in treatment, which might confound the differences between the two groups. Both of these issues are directly related to a lack of randomization procedures applied to assign an intervention. To determine if our findings are robust in face of these limitations, future studies should aim to apply random group allocation.

### 4.2 Implications for program development

DELTA may be expanded toward smoking cessation as well, considering that neither DELTA nor other comparable programs specifically focus on nicotine use disorder or smoking behavior although the vast majority of adolescent SUD outpatients smoke (29). Our study features several elements of pragmatic trials (e.g., diverse settings, simplified analysis design, non-randomization, uncontrolled environments) (30). It is therefore necessary to replicate our findings in multicenter randomized clinical trials controlling for variables relating to therapy setting, adjunct interventions or sample characteristics. For example, showing that DELTA is both feasible and effective when conducted in inpatient settings might extend existing detoxification treatments. Furthermore, DELTA seems to be not sufficient in reducing symptoms of comorbid PTSD, prodromal psychotic symptoms or attentional deficits. Thus, further developments should take into account that on one hand patients with these symptoms need additional treatment in form of disorder-specific therapy and on the other hand DELTA might need to be extended to provide appropriate care for this population.

### 4.3 Implications for similar research studies

Similar evaluation studies could benefit from applying improved strategies to retain and assess adolescent outpatients with SUD. First, providing additional reimbursements and adhering to a fixed contact schedule during the intervention period and the follow-up period may raise the number of participants adhering to intervention sessions and follow-up appointments. However, low adherence to treatment and to research appointments is not uncommon in this population. A comparable feasibility study of *N* = 41 adolescent inpatients with SUD who received a mindfulness-based group intervention with 12 sessions reported a mean of 5.98 sessions out of 12 possible sessions (31). From a clinical standpoint, every realized appointment may be a sign of treatment motivation, while missing single appointments is neither uncommon nor a sign that the SUD does not need to be treated. Additionally, future studies should intent to systematically investigate how SUD specific treatments can and cannot support the reduction of co-occurring symptoms of other disorders.

## 5 Conclusion

We presented first findings that the DELTA intervention for adolescents with SUD is viable, and preliminary results that DELTA might reduce SUD severity. After a future replication, DELTA might be used (a) in those adolescents who do not need to undergo inpatient detoxification treatment or (b) as a supplement following detoxification, helping to stabilize patients in their abstinence once they finish detoxification. Until then, clinical efficacy should be cautiously interpreted.

## Data availability statement

The datasets presented in this article are not readily available because original data is part of the ongoing study. Any publication of raw data has to be permitted by the funding agency. Requests to access the datasets should be directed to lukas.basedow@uni-marburg.de.

## Ethics statement

The studies involving humans were approved by Institutional Review Board, University Hospital C.G.C Dresden (EK 66022018). The studies were conducted in accordance with the local legislation and institutional requirements. Written informed consent for participation in this study was provided by the participants’ legal guardians/next of kin.

## Author contributions

LB: Data curation, Formal Analysis, Investigation, Validation, Visualization, Writing – original draft, Writing – review & editing. SK-P: Formal Analysis, Investigation, Resources, Validation, Writing – original draft, Writing – review & editing. MW: Validation, Visualization, Writing – review & editing. VR: Project administration, Resources, Supervision, Writing – review & editing. YG: Conceptualization, Funding acquisition, Project administration, Resources, Supervision, Validation, Writing – review & editing.
