# Supplementary material for: Evaluation of the multimodal DELTA therapy for adolescents with substance use disorders: an exploratory pilot trial
Source: Front Psychiatry. 2024 Jan 3;14:1284342. doi: 10.3389/fpsyt.2023.1284342 (PMC10793107; doi:10.3389/fpsyt.2023.1284342)
Supplement: Supplementary file 1 [file Table_1.docx]

Supplementary Material

# Supplementary Figures and Tables

*Table S1.* CONSORT Checklist extension for pragmatic trials

| Section | Item | Description of item | Place in text |
| --- | --- | --- | --- |
| Title and abstract | 1 | How participants were allocated to interventions (eg, “random allocation,” “randomised,” or “randomly assigned”) | Title page |
| **Introduction** |  |  |  |
| Background | 2 | Describe the health or health service problem that the intervention is intended to address and other interventions that may commonly be aimed at this problem | Introduction section |
| **Methods** |  |  |  |
| Participants | 3 | Eligibility criteria should be explicitly framed to show the degree to which they include typical participants and/or, where applicable, typical providers (eg, nurses), institutions (eg, hospitals), communities (or localities eg, towns) and settings of care (eg, different healthcare financing systems) | Methods section |
| Interventions | 4 | Describe extra resources added to (or resources removed from) usual settings in order to implement intervention. Indicate if efforts were made to standardise the intervention or if the intervention and its delivery were allowed to vary between participants, practitioners, or study sites | Methods section |
|  |  | Describe the comparator in similar detail to the intervention | Methods section |
| Objectives | 5 | Specific objectives and hypotheses | Methods section |
| Outcomes | 6 | Explain why the chosen outcomes and, when relevant, the length of follow-up are considered important to those who will use the results of the trial | Methods section |
| Sample size | 7 | If calculated using the smallest difference considered important by the target decision maker audience (the minimally important difference) then report where this difference was obtained | N/A |
| Randomisation—sequence generation | 8 | Method used to generate the random allocation sequence, including details of any restriction (eg, blocking, stratification) | N/A |
| Randomisation—allocation concealment | 9 | Method used to implement the random allocation sequence (eg, numbered containers or central telephone), clarifying whether the sequence was concealed until interventions were assigned | N/A |
| Randomisation—implementation | 10 | Who generated the allocation sequence, who enrolled participants, and who assigned participants to their groups | Methods section |
| Blinding (masking) | 11 | If blinding was not done, or was not possible, explain why | Methods section |
| Statistical methods | 12 | Statistical methods used to compare groups for primary outcomes; methods for additional analyses, such as subgroup analyses and adjusted analyses | Methods section |
| **Results** |  |  |  |
| Participant flow | 13 | The number of participants or units approached to take part in the trial, the number which were eligible, and reasons for non-participation should be reported | Figure 1 |
| Recruitment | 14 | Dates defining the periods of recruitment and follow-up | Methods section |
| Baseline data | 15 | Baseline demographic and clinical characteristics of each group | Results section |
| Numbers analysed | 16 | Number of participants (denominator) in each group included in each analysis and whether analysis was by “intention-to-treat”; state the results in absolute numbers when feasible (eg, 10/20, not 50%) | Tables 1,2,3 |
| Outcomes and estimation | 17 | For each primary and secondary outcome, a summary of results for each group and the estimated effect size and its precision (eg, 95% CI) | Tables 1,2,3 |
| Ancillary analyses | 18 | Address multiplicity by reporting any other analyses performed, including subgroup analyses and adjusted analyses, indicating which are prespecified and which are exploratory | N/A |
| Adverse events | 19 | All important adverse events or side effects in each intervention group | N/A |
| **Discussion** |  |  |  |
| Interpretation | 20 | Interpretation of the results, taking into account study hypotheses, sources of potential bias or imprecision, and the dangers associated with multiplicity of analyses and outcomes | Discussion section |
| Generalisability | 21 | Describe key aspects of the setting which determined the trial results. Discuss possible differences in other settings where clinical traditions, health service organisation, staffing, or resources may vary from those of the trial | Discussion section |
| Overall evidence | 22 | General interpretation of the results in the context of current evidence | Discussion section |

*Table S2.* Comparison between participants reached at FU and Non-responders (‘lost’)

|  | **Groups** | | **Group differences** | | |
| --- | --- | --- | --- | --- | --- |
|  | **Total FU *(n* = 67)** | **Total lost *(n* = 79)** | **Test statistic (*df*)** | ***p*** | **Effect size** |
| Females, *n* (%) | 30 (44.7%) | 26 (32.9%) | X^2^ (1) = 2.16 | .142 | *d* = 0.25 |
| Age in years, *M* (*SD*) | 16.2 (1.1) | 16.1 (1.3) | *t* (144) = - 0.20 | .421 | *d* = - 0.03 |
| DUDIT, *M* (*SD*) | 19.4 (11.4) | 16.4 (9.7) | *t* (109) = - 1.50 | .069 | *d* = - 0.29 |
|  | **DELTA FU *(n* = 41)** | **DELTA lost *(n* = 44)** | **Test statistic (*df*)** | ***p*** | **Effect size** |
| Females, *n* (%) | 20 (48.8%) | 15 (34.1%) | X^2^ (1) = 1.89 | .169 | *d* = 0.31 |
| Age in years, *M* (*SD*) | 16.3 (1.0) | 16.0 (1.4) | *t* (83) = - 1.11 | .136 | *d* = - 0.24 |
| DUDIT, *M* (*SD*) | 21.0 (11.1) | 15.7 (9.5) | *t* (62) = - 2.07 | .021 | *d* = - 0.52 |
|  | **WL FU *(n* = 26)** | **WL lost *(n* = 35)** | **Test statistic (*df*)** | ***p*** | **Effect size** |
| Females, *n* (%) | 10 (38.5%) | 11 (31.4%) | X^2^ (1) = 0.33 | .568 | *d* = 0.15 |
| Age in years, *M* (*SD*) | 16.0 (1.2) | 16.3 (1.1) | *t* (59) = 1.08 | .142 | *d* = 0.28 |
| DUDIT, *M* (*SD*) | 17.0 (11.5) | 17.4 (10.0) | *t* (45) = 0.12 | .453 | *d* = 0.04 |

Note: *DUDIT, Drug Use Disorder Identification Test; WL, waitlist condition*

*Table S3.* Associations between changes in primary/secondary outcomes and the number of DELTA sessions in the DELTA subsample, with medium or large associations considered relevant here.

|  | **DELTA participants (*N* = 41)** | | |  | |
| --- | --- | --- | --- | --- | --- |
| **Mean change (from baseline to FU) regarding** | ***N*** | ***p*_two-sided_** | **Effect size *r*_Pearson_** | **Interpretation according to Cohen (1988)** | |
| *Primary outcomes* |  |  |  |  | |
| DUDIT score | 16 | .586 | - .15 | Small/irrelevant | |
| DUDIT-C score | 14 | .752 | - .09 | Small/irrelevant | |
| Nicotine QF | 26 | .128 | - .31 | Medium-sized association, n.s. | |
| Alcohol QF | 15 | .258 | - .31 | Medium-sized association, n.s. | |
| Cannabis QF | 18 | .291 | - .26 | Small/irrelevant | |
| MDMA QF | 8 | .748 | - .14 | Small/irrelevant | |
| Amphetamine QF | 5 | .198 | - .69 | Large association, n.s. | |
| Methamphetamine QF | 4 | .327 | - .67 | Large association, n.s. | |
| *Secondary outcomes* |  |  |  |  | |
| BDI-II sum | 17 | .659 | + .11 | Small/irrelevant | |
| YSR anxious/depressive | 16 | .394 | - .22 | Small/irrelevant | |
| YSR social withdrawal | 16 | .208 | - .33 | Medium-sized association, n.s. | |
| YSR aggressive | 16 | .523 | + .17 | Small/irrelevant | |
| YSR dissocial | 16 | .223 | - .32 | Medium-sized association, n.s. | |
| UCLA symptoms intrusion | 12 | .510 | - .01 | Small/irrelevant | |
| UCLA symptoms avoidance | 12 | .614 | - .05 | Small/irrelevant | |
| UCLA symptoms hyperarousal | 12 | .592 | - .17 | Small/irrelevant | |
| *Exploratory analysis* |  |  |  |  | |
| YSR attention | 16 | .163 | - .36 | Medium-sized association, n.s. | |
| PQ16 sum | 12 | .922 | - .03 | Small/irrelevant | |
| SWLS sum | 14 | .512 | - .19 | Small/irrelevant | |
| *Note:* Amphetamine not included as it was not reported by DELTA participants. *P*-values were not corrected for multiple testing given that differences were not statistically significant in the first place. Interpretation according to Cohen (1988): small (*r* ≥ .10), medium (*r* ≥ .30), large (*r* ≥.50).  ADD/ADHD, attention-deficit disorder with/without hyperativity. BDI-II, Beck Depression Inventory II. DUDIT, Drug Use Disorders Identification Test. MDMA, 3,4-Methyl​enedioxy​methamphetamine. n.s., not significant with *p*_two-sided_ (uncorrected) ≥ .05. PQ16, Prodromal Questionnaire. PTSD, post-traumatic stress disorder. SWLS, Satisfaction With Life Scale. UCLA,UCLA PSTD questionnaire. YSR, Youth Self Report questionnaire. | | | | |  |


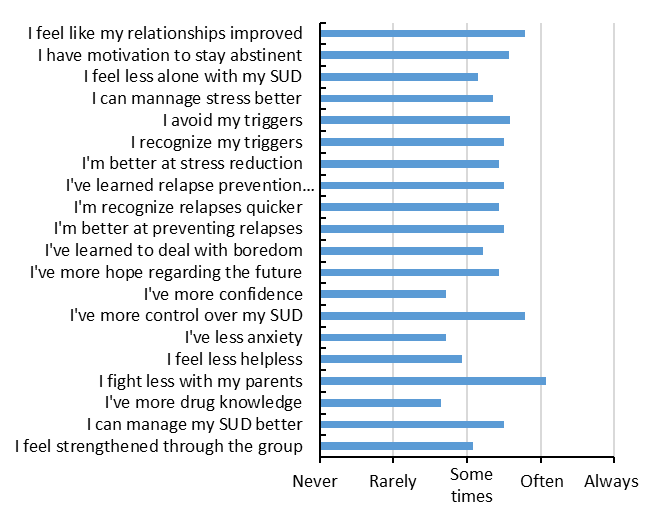


Figure S1*. Subjective ratings of how much the DELTA sessions helped to achieve certain goals in daily life.* N *= 12 to 14 per item.*

References

Cohen, J. (1988). *Statistical power analysis for the behavioral sciences* (2. ed., reprint). Psychology Press.
